# From Data to Knowledge: Systematic Review of Tools for Automatic Analysis of Molecular Dynamics Output

**DOI:** 10.3389/fphar.2022.844293

**Published:** 2022-03-10

**Authors:** Hanna Baltrukevich, Sabina Podlewska

**Affiliations:** ^1^ Maj Institute of Pharmacology, Polish Academy of Sciences, Kraków, Poland; ^2^ Faculty of Pharmacy, Chair of Technology and Biotechnology of Medical Remedies, Jagiellonian University Medical College in Krakow, Kraków, Poland

**Keywords:** molecular dynamics, machine learning, structure-based drug design, clustering, data dimensionality reduction, interaction fingerprints

## Abstract

An increasing number of crystal structures available on one side, and the boost of computational power available for computer-aided drug design tasks on the other, have caused that the structure-based drug design tools are intensively used in the drug development pipelines. Docking and molecular dynamics simulations, key representatives of the structure-based approaches, provide detailed information about the potential interaction of a ligand with a target receptor. However, at the same time, they require a three-dimensional structure of a protein and a relatively high amount of computational resources. Nowadays, as both docking and molecular dynamics are much more extensively used, the amount of data output from these procedures is also growing. Therefore, there are also more and more approaches that facilitate the analysis and interpretation of the results of structure-based tools. In this review, we will comprehensively summarize approaches for handling molecular dynamics simulations output. It will cover both statistical and machine-learning-based tools, as well as various forms of depiction of molecular dynamics output.

## Introduction

Structure-based drug design is becoming an indispensable part of virtual screening campaigns, due to the expanding possibilities of carrying out experiments from this path. It is related both to the achievements in the field of crystallography (expressed by the increasing number of deposited crystal structures), but also to the availability of the computational power and more efficient computational algorithms. Structure-based tools, with their key representatives—docking and molecular dynamics simulations–are a great source of information on the possible interaction schemes occurring between ligand and target receptors (Yang, 2014; Wang et al., 2018).

Molecular docking is a technique that aims to predict the optimal binding mode(s) of a ligand in the respective receptor (Morris and Lim-Wilby, 2008; Guedes et al., 2014; Ferreira et al., 2015). As the docking methodology relies on minimizing free energy of the ligand-receptor complex, the obtained structure can constitute a good starting point for more detailed analysis of ligand-protein interactions during molecular dynamics (MD) simulations (Santos et al., 2019; Wang et al., 2019). Moreover, as most docking tools provide limited flexibility of the target, MD can explore conformational space and generate an ensemble of receptor conformations, which could further be used during screening of chemical databases (Amaro et al., 2018; Acharya et al., 2020). The so-called ensemble sampling has not only increased the hit rate and, thus, improved the quality of virtual screening, but has also allowed efficient docking to the so-called “difficult protein targets” (Fu et al., 2014; Ellingson et al., 2015; Uehara and Tanaka, 2017; Bhattarai et al., 2020).

**FIGURE 1:**
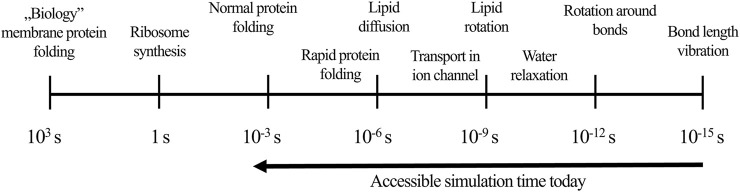
The influence of simulation time on events occurring during MD (according to Lindahl, 2008).

MD is an approach that relies on simulating dynamical changes of the system and capturing its evolution in time. MD offers an insight into the movement of the ligand-receptor complex at an atomistic level. Furthermore, it enables quantitative estimation of parameters that cannot be established in wet-lab experiments, e.g., values of torsional angles to describe flexibility, solvent accessible surface area to predict stability, or change in the entropy for distinct structures, such as water molecule in particular location (Ferreira et al., 2015; Leimkuhler and Matthews, 2016; Hollingsworth and Dror, 2018). The basis of the classical MD methodology is solving the Newton’s motion equations for each atom in the system, where the potential energy and forces of interacting particles are from the force-field definitions (Sutmann, 2002; Lindahl, 2008). These approximations are necessary to balance between the required accuracy and optimal speed of simulations’ performance. Moreover, MD timestep should be very small—1–10 fs – in order to minimize errors related to the potential energy estimation (Binder et al., 2004; Leimkuhler and Matthews, 2016). Huge numbers of timesteps, which are required for even relatively short simulations, contribute to the consumption of a great amount of computational resources. Fortunately, due to the increasing computational power and possibilities to perform simulations with the use of graphical processing units (GPU), MD simulations reached a millisecond time scale allowing to investigate events such as protein folding (Figure 1; Lindahl, 2008).

Thus, the amount of data produced by MD has dramatically increased over recent years and is far beyond the accessibility of manual analysis. For this reason, it is crucial to develop automatic tools for post-processing of such data. Great numbers of approaches are offered specifically by the software for MD simulations. Nevertheless, a lot of new independent methods for automated analysis have appeared recently, which are based on various statistical methods and machine learning (ML).

ML approaches are nowadays used at each stage of the drug design process and development (Ballester, 2019; Vamathevan et al., 2019; Patel et al., 2020). Their most common application involves the evaluation of compound potential bioactivity in ligand-based virtual screening (Melville et al., 2009; Carpenter and Huang, 2018; Hussain et al., 2021); however, they are also widely applied in the evaluation of compound physicochemical and ADMET properties (Göller et al., 2020; Göller et al., 2022; Jia and Gao, 2022). The ML role in computer-aided drug design is not limited to the assessment of compound libraries, but a number of generative approaches is used to enumerate new sets of potentially active compounds (Baskin, 2020). Moreover, ML can help in the compound optimization and indication of features, which are important for a particular type of activity, thanks to the wide range of interpretability tools (Hudson, 2021). ML methods also support structure-based path of virtual screening tasks – they assist in the detection of ligand-protein interaction patterns characteristic for considered activity profiles (Khamis et al., 2015; Khamis and Gomaa, 2015; Khamis et al., 2016), as well as in the detection of complex relationships between ligand-protein interaction schemes occurring during MD simulations (Podlewska et al., 2020; Kucwaj-Brysz et al., 2021).

In this review, we comprehensively summarize existing approaches to automatic handling of MD simulations’ outputs. We will describe approaches available within the MD software, but our main focus is on the automatic statistical and ML-based post-processing tools.

## Tools Available Within the MD Software or Packages Dedicated To MD Output Analysis

Numerous software packages are able to perform MD simulations. The list of the most popular programs includes GROMACS (Abraham et al., 2015), HyperChem (Laxmi and Priyadarshy, 2002), AMBER (Case et al., 2005), LAMMPS (Thompson et al., 2021), CHARMM (Brooks et al., 2009), DL_POLY (Todorov et al., 2006), HOOMD (Glaser et al., 2015), TINKER (Lagardère et al., 2018), NAMD (Phillips et al., 2005), and Desmond (Bowers et al., 2006). The resulting simulation trajectory can then be analyzed at different levels – from the qualitative visualization of changes occurring in the modeled system to detailed investigation of variations in atom positions and ligand-protein interactions. Due to the high amount of data produced during MD simulations (of up to several terabytes size), programs for MD analysis should also be able to efficiently deal with such data volumes.

The list of the most known packages for MD simulations analysis opens VMD [Visual Molecular Dynamics (Humphrey et al., 1996)], developed by the Theoretical and Computational Biophysics Group at the University of Illinois at Urbana-Champaign. VMD is a program designed for interactive visualization and analysis of biomolecular systems including processing of very large systems (composed of up to billion particles). The software is written in C and C++ (source code available) and is distributed free of charge. Convenient graphical interface supports performing various types of coordinate analysis on Unix, MacOS, and Windows operating system, along with NVIDIA OptiX and CUDA support. In addition to the built-in analysis tools applicable to trajectories processing, VMD has a broad collection of plugins and scripts (VMD Plugin Library, 2021, n. d.; VMD Script Library (2021), n. d.).

Execution of Tcl and Python scripts and implementation of developed plugins enables adjustement of VMD capabilities to users’ needs without recompiling the source code. Both types of tools are distributed under an open-source license, unless otherwise stated. Moreover, researchers are encouraged to develop and share new utilities in order to support the growth of the VMD community and development of the software. VMD plugins are divided into the “molfile” plugins, which enable working with multiple file formats of molecular data, and scripting extensions used to perform requested tasks. Plugins dedicated to data analysis allow performing various calculations: from RMSD (*RMSD Tool*, *RMSD Trajectory Tool*) to electrostatic potentials (*APBSRun*, *Delphi Force*) and IR spectral density (*IRSpecGUI*). Resulting outcomes can be visualized through generated plots—*GofRGUI*, *NAMD Plot*, *RamaPlot*, *Timeline*—or as maps—*Contact Map*, *VolMap*, *HeatMapper*, *PMEpot*. There are also plugins capable of analysing free-energy perturbation calculations (*AlaScan*, *ParseFEP*) and obtaining data on proteins—*Intervor* (extracts and displays protein-protein interface), *SurfVol* (measures surface area and volume of proteins), and *NetworkView* (shows protein interaction networks). Developed statistical tools visualize clusters of structure conformations (*Clustering Tool*) or perform normal mode visualization and comparative analysis (*NMWiz*). VMD has constantly been developed: the latest version (1.9.3) includes introduction of the following major features: introduction of new QwikMD plugin connecting VMD with MD program NAMD, enabling quick preparation of common molecular simulations; the TopoTools plugin used for automated topology conversion from CHARMM to GROMACS: the new TachyonL-OSPray ray tracing engine for generating high quality renderings of molecular systems containing hundreds millions of particles; and OpenGL rendering for parallel visualization runs on “headless” clouds and petascale computers.

PTRAJ (Process TRAJectory) is another example of a tool enabling post-processing of MD data (Roe et al., 2013). It was dedicated for the analysis of the AMBER output. Its successor, CPPTRAJ, emerged as a response to the growing trajectory sizes, offering a wider range of functionalities and more efficient data processing. In contrast to PTRAJ (written primarily in C), CPPTRAJ code is based on C++ and the whole program structure was reorganized to facilitate the addition of new functionalities. The programs and their source code are freely available under the GNU General Public License version 3 and are distributed within the AmberTools21. The strong point of CPPTRAJ is batch-processing, which allows the use of remote sites for analysis and possibility of combining various types of commands, trajectories, and topologies in the same run. Other important features of CPPTRAJ are: the availability of MPI, OpenMP, and CUDA parallelization, support for implementation of variables and loops, and possibility to apply atom masking to specify which part of the system should be analyzed. The number of developed commands applicable for MD data analysis is great, including simple calculations, such as estimation of the number of hydrogen bonds (*hbond*), and multiple examples of more complex tools, such as performing non-linear curve fitting (*curvefit, multicurve*) and linear regression (*regress*), matrix based calculations (*crosscorr, diagmatrix, hausdorff, modes*), estimating auto-/cross-correlation (*autocorr, correlationcoe, timecorr*), creating histograms (*hist, kde, multihist*), and many more (Case et al., 2021). CPPTRAJ development has resulted in new features, among which are: rewritten code expanding clustering capabilities, ability to RMS-fit grids onto coordinates, automatic calculation of multiple puckers, speeding up the non-bonded energy calculation, enhancing the performance of the *permutedihedrals* and *randomizeions* commands, and automation of downloading and building external libraries in CPPTRAJ (2021).

MDAnalysis is an object-oriented library developed for the analysis of MD trajectories and protein structures (Michaud-Agrawal et al., 2011). The package is written in Python and Cython and uses NumPy arrays to expand its functionality. MDAnalysis is available under the GNU General Public License version 2.0 (https://github.com/MDAnalysis/mdanalysis). The analysis modules are capable of assessing distances and contacts (e.g., calculating path similarity, which reveals geometric similarity of trajectories useful for identification of patterns in trajectory), performing dimensionality reduction and carrying out volumetric analysis (e.g., linear density estimation). Other modules analyze the structure of macromolecules (such as HELANAL (Sugeta and Miyazawa, 1967; Bansal et al., 2000)—a tool for the analysis of protein helices), polymers (including determination of the polymer persistence length), nucleic acids and, finally, membrane and membrane proteins (namely, HOLE (Stelzl et al., 2014), a suite of tools used to assess pore dimensions of the holes as a function of time). Recently MDAnalysis announced the introduction of a command-line interface in answer to user needs, and a number of supported analysis modules is provided in the documentation.

MDTraj (McGibbon et al., 2015) is a Python library applied for MD trajectory manipulation and analysis, whose goal is to provide interafce between MD data and modern tools and programs for statistical analysis and visualization based on Python. MDTraj is licensed under the Lesser GNU General Public License (LGPL v2.1+) on GitHub (https://github.com/mdtraj/mdtraj). MDTraj works with every possible MD data format, focusing on speed and efficient performance and providing multiple analysis possibilities. Available functions identify hydrogen bonds, compute distances to create residue-residue contact maps, assess secondary structure of the protein and assign code according to the implemented Dictionary (Kabsch and Sander, 1983), calculate solvent-accessible surface area (SASA) and NMR scalar coupling, as well as determine nematic order parameters, which describe the orientational order of a system from 0 to 1. Another special feature is the particularly fast RMSD computations due to performance optimization based on Haque at al. (2014) along with C/C++ code implementation. Moreover, MDTraj documentation gives access to 14 notebooks containing analysis examples with executable code—e.g., PCA with scikit-learn ML library followed by plotting data using Matplotlib.

LOOS (Lightweight Object-Oriented Structure-analysis) (Romo et al., 2014; Grossfield and Romo, 2021) aims at enabling rapid development and testing of new tools for MD analysis. Additionally, the program includes a number of easy-to-use prebuilt applications. As LOOS is a C++ library, its combination with Python interface (PyLOOS) resulted in high performance and simplicity of use and further development. Moreover, the C++ layers could be used independently for even more efficient utilization of resources. LOOS is freely distributed under the GPLv3 license and is available via GitHub (https://github.com/GrossfieldLab/loos). In LOOS, 140 prebuilt tools are grouped into the following categories: macromolecule tools (e.g., computation of the radial distribution function), hydrogen bonding handling, principal component analysis (PCA), elastic network models (ENM), clustering, assessment of statistical error (e.g., block-averaged standard error calculations), and convergence. The tools included in the "membrane systems" category are dedicated for analyzing lipid bilayers and associated systems (e.g., calculation of molecular order parameters. Furthermore, 2D Voronoi decomposition tools are used to obtain data within a particular membrane slice. 3D density distributions tools generate 3D histograms from MD trajectories. They were originally created for visualization of water distribution; however, they are able to estimate membrane lipid density as well.

Pteros (Yesylevskyy, 2012; Yesylevskyy, 2015) is a high-performance molecular modeling library available for C++ and Python. It lets users analyse MD data and develop new analysis tools with the assistance of the easy-to-use APIs in both of the above-mentioned programming languages. In order to accelerate the analysis process, Pteros asynchronously reads files with MD trajectories and performs analysis tasks in parallel. Analysis plugins are completely independent and, besides typical calculations, provide more specific manipulations. For example, they enable assessing properties related to curvature with the Curvature plugin, which computes mean and Gaussian curvatures of various lipid aggregates, smooths membrane surfaces, and calculates other properties of molecules embedded into the lipid membrane. While the above-mentioned plugin is not open-source, Pteros is a free software distributed under Artistic License and available at GitHub (https://github.com/yesint/pteros).

Till now, we have described exclusively open source software and libraries, which serve as powerful and freely available tools for MD output analysis. Nevertheless, some commercial software is also worth mentioning, e.g., Molecular Operating Environment (MOE) [Molecular Operating Environment (MOE), 2019], Desmond (*Schrödinger Release 2021*–4: Desmond Molecular Dynamics System, 2021), and CHARMM (Brooks et al., 2009). MOE constitutes a platform for integrated computer-aided molecular design with vast capabilities: QSAR models generation, virtual screening, protein engineering, homology modeling, as well as carrying out MD simulations. However, MOE offers limited opportunities for MD analysis, as only Free Energy Calculations along with Torsion Scan and Analysis are mentioned at the official software webpage. Greater analysis possibilities are provided by Desmond—a commercial software available without cost for non-commercial use, developed by D. E. Shaw Research for high-speed MD simulations of biological systems. Desmond offers multiple panels for different post-processing operations, such as Trajectory Frame Clustering Panel, Simulation Quality Analysis Panel (enabling estimation of potential energy, temperature, pressure, etc.), Simulation Event Analysis Panel (enabling calculation of geometric and energy-based properties, e.g., RMSF, hydrogen bonds, Coulomb energy), and Radial Distribution Function Panel. What is more, Desmond provides distinct panels for metadynamics and replica exchange simulations analysis, and Python scripts applicable for PCA, density profile calculations, and others. The advantages of MD data analysis in Desmond are its detailed tutorials, intuitive GUI, and conveniency of some tools, such as Simulation Interaction Diagram. Its output is saved as a pdf file, which contains results of protein-ligand system analysis in the form of colored plots, together with the short explanation of the meaning of each calculated property.

Plenty of other software and tools are useful in MD data analysis; among them are GROMACS (Abraham et al., 2015) and CHARMM (Brooks et al., 2009)— well-known MD programs capable of performing analysis tasks as well. Carma (Glykos, 2006) is a lightweight program written in C along with its graphical user interface grcarma (Koukos and Glykos, 2013) and Wordom (Seeber et al., 2007; Seeber et al., 2011) - a simple and fast command-line utility. MMTSB (Feig and Karanicolas, 2004) is a set of tools for enhanced sampling and multiscale molecular modeling approaches, while Simulaid (Mezei, 2010) is a program for carrying out analysis tasks of multiple types and MD trajectory data manipulation. MMTK (Hinsen, 2000), the Molecular Modeling Toolkit, contains MD analysis scripts; both Bio3D package (Grant et al., 2006) written in R language, and Python toolkit. MD-Tracks (Verstraelen et al., 2008) provides statistical analysis of MD data, and ST-Analyzer (Jeong et al., 2014) is an intuitive and simple web-based GUI environment, with nine analysis modules for extraction of various parameters from MD output.

## Machine Learning—Classes of Models Used in the Structure-Based Drug Design

ML methods have become an integral element of structure-based path of drug design, and they assist in the analysis of both docking and MD simulations (Dutta and Bose, 2021). The general task of ML is to detect relationships and complex patterns in large datasets. As the amount of data produced in the structure-based path has recently grown enormously, the application of ML methods for MD outcome analysis is becoming more and more popular. Within ML methods, we can also distinguish deep learning (DL) algorithms with their main usage in computer-aided drug design to generate examples of new potential ligands via generative approaches.

The most popular classes of ML models applied in the broadly understood campaigns for searching for new drugs include:

1) Bayesian models—a collection of models based on the Bayes’ theorem. It defines the probability of an event on the basis of prior knowledge of conditions, which might be influencing this event. The Bayes’ theorem in its simplest form (taking into account only two events, A and B) can be described using the following equation:

$$P\left(A\text{|}B\right)=\frac{P\left(B\text{|}A\right)P\left(A\right)}{P\left(B\right)},$$

where P (A|B) is a conditional probability of occurrence of event A, given that B is true; P(B|A) is a conditional probability of occurrence of event B, given that B is true; and P(A) and P(B) are probabilities of occurrence A and B, respectively, without any conditions (P(B) > 0).

Bayes’ theorem for a higher number of events adopts the following form:

If B, T_1_,…,T_n_ are such events that:

P(B) > 0, $B\text{C}\cup_{i=1}^{n}T_{i}$ and $T_{i}\cap T_{j}=\phi \left(i\neq j\right),$ then:

$$P\left(T_{j}\text{|}B\right)=\frac{P\left(B\text{|}T_{j}\right)P\left(T_{j}\right)}{\sum_{i=1}^{n}P\left(B\text{|}T_{i}\right)P\left(T_{i}\right)}.$$

In drug design approaches, Bayes’ theorem is most often used within the Naïve Bayes algorithm. In such a case, Bayes’ theorem is used together with an assumption of events (features) independence (Berrar, 2019).

Another concept using Bayes’ theorem is Bayesian statistics, in which all observed and unobserved parameters of a statistical model are given a joint probability distribution (prior and data distribution). Bayesian statistics expresses probability as a *degree of belief*, and Bayes’ theorem is used to assign a probability distribution to quantitatively describe this *degree of belief* in the form of a set of parameters (van de Schoot et al., 2021).

The Bayesian concept is also used in fuzzy clustering (Glenn et al., 2015).

**FIGURE 2:**
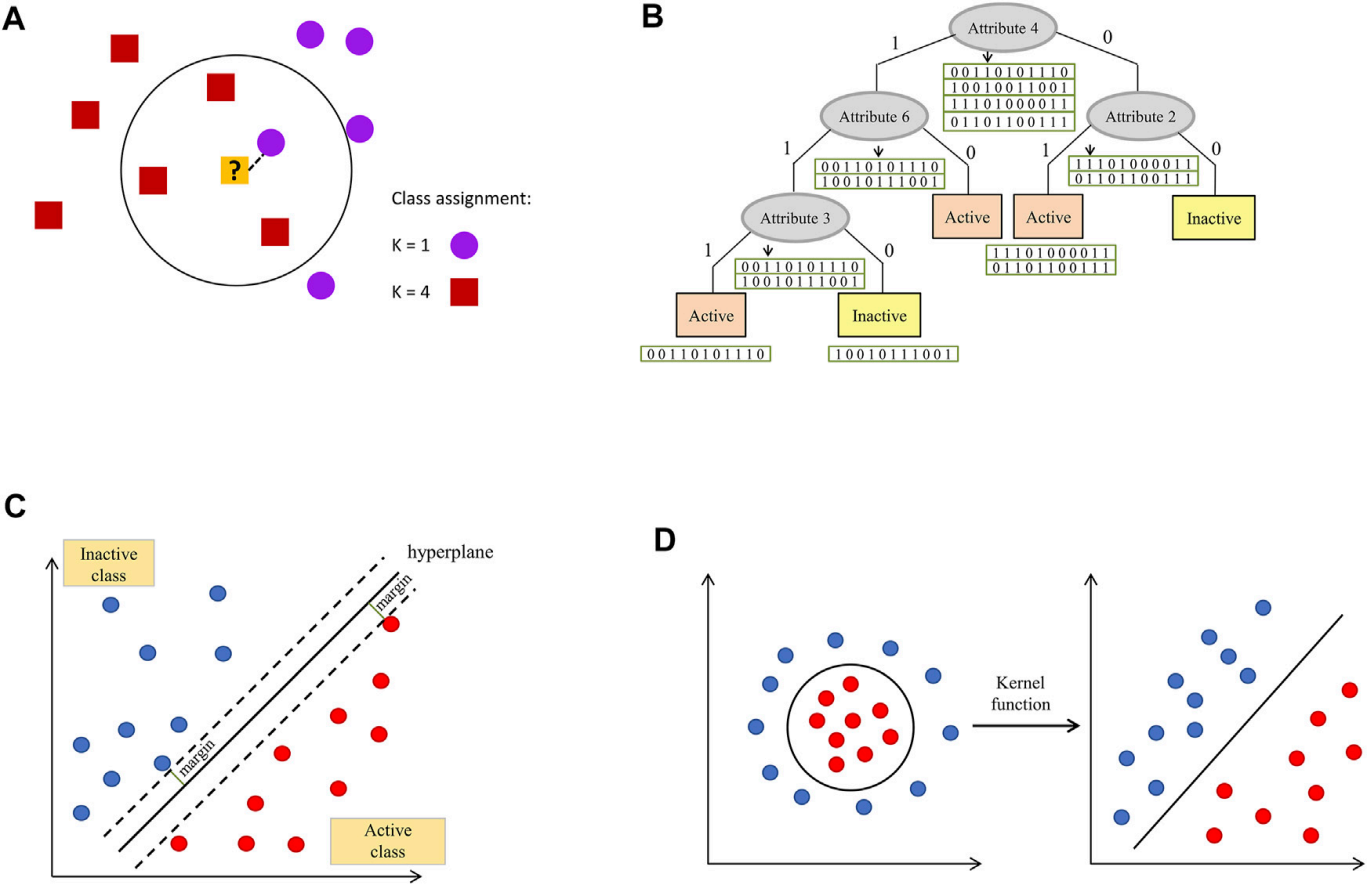
Visualization of selected ML algorithms: **(A)** k-nearest neighbor, **(B)** decision tree, **(C)** hyperplane with the highest separation margin constructed within the support vector machines algorithm implementation, **(D)** data transformation to the space, in which they are linearly separable with the use of the kernel function.

2) K-nearest neighbors methods – based on the determination of distances between an evaluated sample and representatives of the training set. In its simplest form (K = 1), the evaluated sample is assigned to the class of its closest neighbor from the training set (or value of the considered parameter of the closest neighbor is returned in the case of regression). If a higher number of examples closest to the query is considered (K > 1), voting for the most frequent class label is carried out (classification) or values of evaluated parameters are averaged (regression)–Figure 2 a (Cover and Hart, 1967; Hall et al., 2008).

In MD studies, k-nearest neighbors algorithm is also used in clustering procedures aimed at the formation of groups of geometrically similar conformations (Keller et al., 2010).

3) Trees—tree-based algorithms are considered to be one of the most efficient and most broadly used types of ML models. Their important advantage is their simplicity and ease of interpretation, which play a role in drug design protocols (e.g., by the possibility of indication of features important for a particular compound activity). Predictions can be made using one decision tree or multiple tress (as it is in the case of Random Forest). Attributes for a root and subsequent nodes are selected on the basis of their discrimination power (at each level, a feature which provides the best distinguishment between considered classes is selected). Evaluation of new examples is carried out via checking values of features present in the subsequent nodes -Figure 2B (Breiman et al., 1984; Quinlan, 1986).
4) Neural networks—neural networks search for relationships in data in such a way that they mimic the processes occurring in the human brain. Their neurons are constituted by a mathematical function, which collects and classifies information. Such artificial neurons are interconnected (such connections reflect biological synapses, called edges) and they have the ability to communicate with each other. A neuron (node) receives a signal, processes it, and passes the respective information to the connected neurons. Typically, neurons are organized into layers, and the signal is passed from the input layer (the first one) to the output layer (the last one) (Hopfield, 1982).

A special type of neural network that has recently gained enormous popularity is deep neural network (DNN) with “deep” referring to the application of multiple layers in the network (LeCun et al., 2015; Schmidhuber, 2015).

Neural networks concept is also applied in unsupervised approaches for MD data clustering, e.g., in the form of Self Organizing Maps (SOMs) (Hyvönen et al., 2001; Fraccalvieri et al., 2013; Mallet et al., 2021). In order not to lose the topological properties of the input space, a neighborhood function is used.

5) Support Vector Machines (SVM)—an algorithm according to which each data item constitutes a point in n-dimensional space (n is equal to the number of features), with coordinates defined by the particular feature value. The task of the model is to find a hyperplane, which discriminates example classes with the highest margin (Figure 2C). As linear discrimination is often not possible, a kernel function needs to be applied in order to transform the input into a space of higher dimension, so an inseparable problem is converted into a separable one–Figure 2D (Cortes and Vapnik, 1995).

## Clustering and Reduction of Data Dimensionality

The most common approach to use the automatic post-processing of the MD simulations output is the reduction of dimensionality and clustering (Amadei et al., 1993; Lange and Grubmüller, 2006).

### Clustering

Clustering, from its assumptions, is an unsupervised technique of finding patterns and relationships in data. In contrast to the previously described techniques, clustering does not require the presence of the training set, as its aim is to form subgroups of similar objects. Clustering algorithms use various “distance” measures to evaluate object similarity. Two main groups of clustering approaches can be distinguished, namely partitional and hierarchical, both of which can be carried out in the bottom-up agglomerative way or using a top-down divisive approach (Kaufman and Rousseeuw, 1990). Another group of data grouping methods are density-based schemes, in which the clusters refer to the peaks of the probability distribution (or free energy minima) from which the data are collected (Sander, 2011; Glielmo et al., 2021). In MD simulations, such probability peaks typically correspond to metastable states of the system. An example application of density-based clustering to the analysis of MD data is density-based spatial clustering of applications with noise (DBSCAN) (Ester et al., 1996; Schubert et al., 2017), in which the clusters are defined as regions with density above the particular threshold. Such an approach was used to find representative structures from MD simulations and analyze MD trajectories (Wang et al., 2013). MD trajectories have also been analyzed by the density peak clustering.

The most popular partitional clustering technique is the K-means algorithm. Clustering in this approach starts from the random placement of K initial centroids. Then, K clusters are formed iteratively in such a way that a point which is closest to a particular centroid is added to the respective cluster, and a new centroid for each cluster is determined. When the cluster membership does not change (the convergence is obtained), the process is stopped. The drawback of K-means clustering is the dependence of the final outcome on the initial choice of the centroids. Problems might also occur when significant variations in the cluster sizes or densities appear, when data outliers are present, or when the ‘natural’ clusters have non-spherical shapes (Hartigan and Wong, 1979; Huang, 1998).

The starting point of agglomerative hierarchical clustering is a formation of singleton clusters from each object from the dataset. Then, iterative linkage of the nearest clusters is carried out, until the whole dataset constitutes one group. On the basis of the resulting dendrogram, the final division of data is produced. Hierarchical clustering is deterministic, but it requires high computational power and storage abilities, which limits its application to small datasets.

The most popular metric used to evaluate MD simulations’ output in terms of data proximity is Root Mean Square Deviation (RMSD). Despite the presence of some drawbacks [e.g., incidents of wrong conclusions when applied to equilibrium evaluation (Grossfield and Zuckerman, 2009)], it is still the most frequently used method for comparison of conformation similarity. Several different solutions were also proposed, such as the application of Euclidean Distances Matrices (EDM) (de Souza et al., 2017); however, they have not gained such wide popularity as RMSD.

### Evaluation of Clustering Approaches

The evaluation of clustering is not easy, as falling into the group of unsupervised approaches, clustering does not refer to true labels. One group of cluster assessment methods is the so-called “internal evaluation,” where clusters are evaluated on the basis of the clustered data. In general, in such an evaluation, the highest scores are assigned to the approaches which produce clusters of high similarity between particular cluster elements and low similarity between elements belonging to different clusters (Rand, 1971). An example of internal measure of clustering quality is Davies-Bouldin index (DB) (Davies and Bouldin, 1979):

$$DB=\;\frac{1}{n}\overset{n}{\underset{i=1}{\sum }}\underset{j\neq i}{\max}\left(\frac{\sigma_{i}+\sigma_{j}}{d\left(c_{i},c_{j}\right)}\right)$$

with n being the number of clusters, *c*_*i*_, c_j_ being centroids of clusters *i* and *j*, respectively; σ_i_ refers to the average distance of elements belonging to cluster i to its centroid c_i_; and d (ci,cj) is the distance between centroids of clusters i and j. The lower the values of DB index, the better they are.

Another approach of the assessment of clustering quality is external evaluation, which refers to pieces of information that were not used during clustering. External evaluation can be based on the known class labels or on some benchmark datasets. However, if the true class labels are known, the clustering is actually not needed (de Souto et al., 2012).

Before the application of methods for clustering evaluation, the dataset should be examined in terms of the clustering tendency. If the dataset is composed of the uniformly distributed points (therefore, there is no clustering tendency present), then the identified clusters may be invalid. In order to verify the clustering tendency, the Hopkins test (Hopkins and Skellam, 1954) can be used (statistical test for spatial randomness of a variable).

### Reduction of Data Dimensionality

Principal Component Analysis (PCA) is an approach for the reduction of the data dimensionality via transformation of a large set of variables into a smaller one, preserving as much information of the original set as possible (Ichiye and Karplus, 1991; Jolliffe, 2002; Jolliffe and Cadima, 2016). The goal is obtained via extraction of important information from the data table and its representation in the form of new orthogonal (linearly independent) variables (principal components). Then, the relationships between observations and variables can be displayed in the form of points in the maps. PCA is based on the assumption that the phenomena of interest can be explained by variances and covariances between original variables from the dataset. PCA is often applied before performing the clustering procedure. In MD-related applications, PCA is responsible for extracting the dominant modes in the molecule motion. It should be pointed out that, during the MD, the Cartesian positions of all atoms of the simulated system (of a size of thousands or even millions of atoms) are recorded in every time step, which indicates the importance of application of post-processing methods. If the dimensionality reduction is carried out properly, all relevant information is preserved, and the analysis of the MD output is valid.

Another approach for reduction of data dimensionality is multidimensional scaling (MDS), which determines the data space of lower dimension with the best possible preservation of the pairwise distances between data points (Young and Householder, 1938; Torgerson, 1952). Its mode of action is closely related to PCA; however, for MDS it is sufficient to provide a pairwise distance between points (their exact positions are not necessary).

PCA and MDS are representatives of linear methods of data dimensionality reduction; however, there is also a number of non-linear approaches to this task, with such examples as isometric features mapping (Tenenbaum et al., 2000), kernel PCA (Schölkopf et al., 1998), diffusion map (Coifman et al., 2005; Coifman and Lafon, 2006), and t-Distributed Stochastic Neighbor Embedding (t-SNE) (van der Maaten and Hinton, 2008). Low-dimensional spaces to embed high-dimensional data are also more and more often determined using DL approaches. One of the most popular DL techniques for reduction of data dimensionality is autoencoder (Kramer, 1991). Autoencoder maps input configuration to representation of lower dimension and then maps it back to the original space *via* respective decoder. Low-dimensional representation is learned *via* minimization of error between the original data points and data points obtained by the application of the above-mentioned decoder. Another DL-based approach for reduction of data dimensionality falls into the group of generative neural networks. Its representatives include Variational Autoencoders (VAEs) (Lopez et al., 2018) and Generative Adversarial Networks (GANs) (Goodfellow, et al., 2014).

### Examples of Clustering and Data Dimensionality Reduction for MD Output Analysis

Unsupervised procedures are widely applied in the MD outcome analysis, due to the above-mentioned problem of the vast amount of data produced during simulations: clustering data into groups gathering similar conformations obtained during MD, and reduction of data dimensionality which lowers the number of features considered. Both these approaches help in the analysis of MD output.

The problem of clustering MD data emerged quite early. The first reports of clustering MD output were released in the early 1990s (Gordon and Somorjai, 1992; Torda and van Gunstered, 1994). Various groups also compared effectiveness of various clustering algorithms (Shao et al., 2007; Keller et al., 2010; Abramyan et al., 2016). Nowadays, clustering of MD data has become a standard procedure applied in order to facilitate interpretation and analysis of MD trajectories (Bruno et al., 2011; De Paris et al., 2015a; De Paris et al., 2015b; Rudling et al., 2018; Takemura et al., 2018; Evangelista et al., 2019; Yoshino et al., 2019; Bekker et al., 2020; Roither et al., 2020; Araki et al., 2021; Mallet et al., 2021; Wu et al., 2021) and new algorithms to improve this procedure are constantly developed.

Dimensionality reduction of MD data with the use of PCA was also first used in the early 90s (Ichiye and Karplus, 1991; Amadei et al., 1993) and since that time its application in MD output analysis has been constantly growing (Das and Mukhopadhyay, 2007; Chiappori et al., 2010; Kim et al., 2010; Casoni et al., 2013; Ng et al., 2013; Novikov et al., 2013; Bhakat et al., 2014; Sittel et al., 2014; Ernst et al., 2015; Chaturvedi et al., 2017; Cossio-Pérez et al., 2017; Fakhar et al., 2017; Chen, 2018; Cholko et al., 2018; An et al., 2019; Barletta et al., 2019; Girdhar et al., 2019; Karnati and Wang, 2019; Lipiński et al., 2019; Martínez-Archundia et al., 2019; Wu et al., 2019; Magudeeswaran and Poomani, 2020; David et al., 2021; Majumder and Giri, 2021). Although PCA is the most popular approach applied to handle MD trajectories, other data dimensionality reduction methods are also used in the MD field. Pisani et al. used MDS to examine conformational landscapes of CDK2 (Pisani et al., 2016) and Bécavin et al. improved the application of MDS for MD data by using singular value decomposition. MDS in the context of MD was also described by Troyer and Cohen (1995), Andrecut (2009), Tribello and Gasparotto (2019), and Srivastava et al. (2020). There are also examples of the application of other approaches: isometric feature mapping (Stamati et al., 2010), kernel PCA (Antoniou and Schwartz, 2011), diffusion map (Rohrdanz et al., 2011; Zheng et al., 2011; Zheng et al., 2013a; Zheng et al., 2013b; Preto and Clementi, 2014), t-SNE (Zhou et al., 2018; Zhou et al., 2019; Spiwok and Kříž, 2020), and VAE (Hernández et al., 2018; Shamsi et al., 2018; Moritsugu, 2021; Tian et al., 2021).

## Markov State Modeling

Markov state modeling (MSM) (Pande et al., 2010; Husic and Pande, 2018) is another approach widely applied in the MD-based studies. MSM can be used to characterize events that occur at longer timescales than available computational power to perform such long simulation. Such MDs are simulated as transitions between a set of discrete stable states. The MSM parametrization can be performed *via* running several short MDs, which can be computed in parallel. The main difficulty in the MSM application is definition of the above-mentioned stable states (Abella et al., 2020). In general, MSM is an approach for modeling random processes with the use of the Markov assumption, which is when the present state is given, all following states are independent of all past states. MSMs describe the stochastic dynamics of a biomolecular system using two objects: a discretization of the high-dimensional molecular state space into n disjoint conformational sets and a model of the stochastic transitions between these states [usually described by a matrix of conditional transition probabilities (Chodera and Noé, 2014)].

Examples of MSM applications in drug design include: examination of the binding kinetics of the trypsin inhibitor benzamidine (Buch et al., 2011), description of the multiple unbinding pathways of ligands dissociating from FKBP (Huang and Caflisch, 2011), examination of substrate binding mechanism of HIV-1 protease (Pietrucci et al., 2009), analysis of binding pathways of opiates to µ-opioid receptors (Barati et al., 2018), reconstruction of binding process of alprenolol to the beta2-adrenergic receptor (Bernetti et al., 2019), membrane-mediated ligand unbinding of the PK-11195 ligand from the translocator protein (TSPO) (Dixon et al., 2021), study of the two bromodomain-inhibitor systems using multiple docked starting poses (Dickson, 2018), examination of the unbinding kinetics of a p38 MAP kinase type II inhibitor (Casasnovas et al., 2017), examination of ligand-induced active-inactive conformation change of beta-2 adrenergic receptor (Bai et al., 2014), and investigation of the interplay of conformational change and ligand-binding kinetics for the serine protease trypsin and its competitive inhibitor benzamidine (Plattner and Noé, 2015).

## Examples of ML-Based Analysis of MD

**FIGURE 3:**
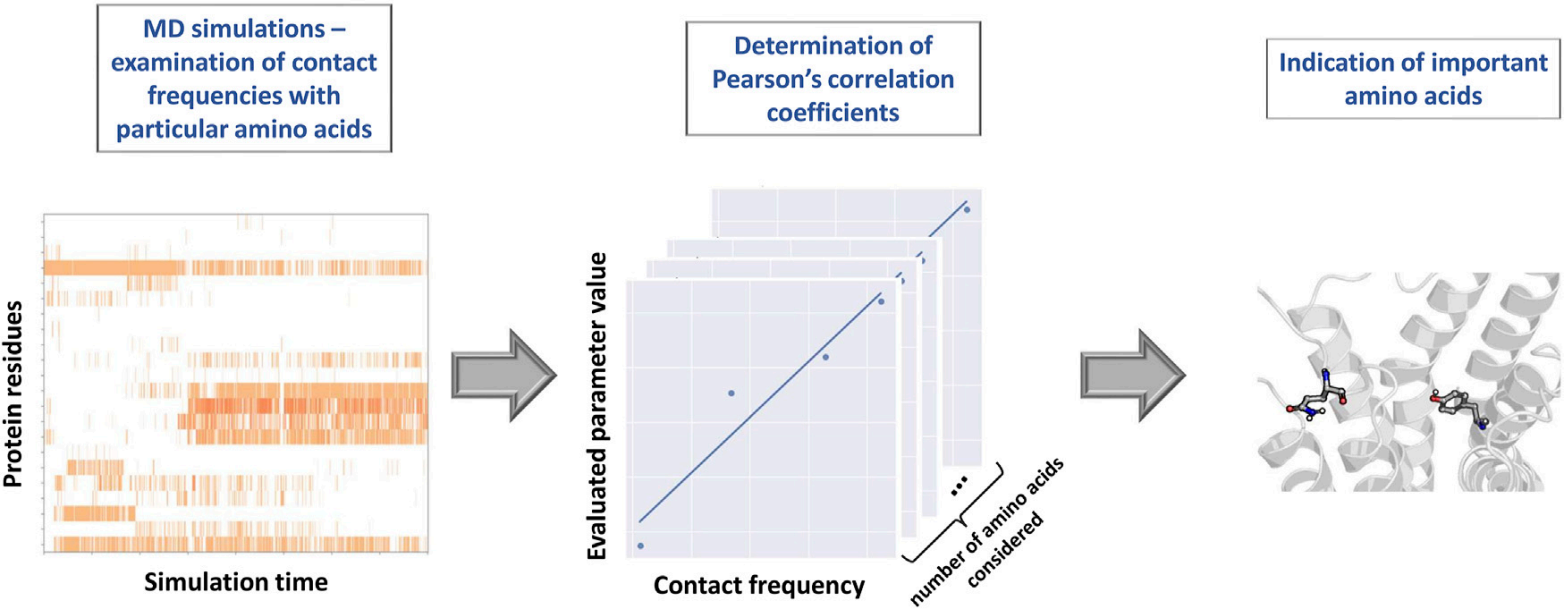
Scheme of the protocol for indication of the important amino acids on the basis of the contact frequency with particular amino acids during MD simulations.

The proper representation of MD outcome opens the door to the wide range of possibilities in terms of the post-processing approaches. Podlewska et al. (2020) and Kucwaj-Brysz et al. (2021) analyzed ligand-receptor contact patterns occurring during MD simulations and examined them with reference to the modeled property. Via the calculation of the Pearson’s correlation coefficient between the contact frequencies and values of examined parameters, the highest correlated residues (considered as the most important for the modeled property) were detected. Scheme of the above-described protocol is presented in Figure 3. At first, each simulation frame was represented with the use of the Structural Interaction Fingerprints (Singh et al., 2006). Then, for each amino acid, the contact frequency during simulation was calculated. Finally, for each protein residue, the Pearson’s correlation coefficients between the respective contact frequency and values of the evaluated compound parameters were determined. The highest correlated positions were indicated as those which should be considered in detail during the further design of compounds of particular activity profile.

Riniker (2017) developed a molecular dynamics fingerprint (MDFP) to combine MD approach with ML methods. MDFPs were obtained *via* the extraction of three properties from MD trajectories: intramolecular and total potential energy of the solute, radius of gyration, and solvent-accessible surface area resulting in a vector of floats. The fingerprint also contained information on the distribution of each property, characterized by its average, standard deviation, and median values. In addition, MDFP was enriched with standard 2D fingerprints: Morgan fingerprints and 2D-counts fingerprints from RDKit (number of heavy atoms, number of rotatable bonds, number of N, O, F, P, S, Cl, Br, and I atoms in the compound). Such representation constituted an input for ML models, which were trained to predict solvation free energies in five different solvents (water, octanol, chloroform, hexadecane, and cyclohexane) and partition coefficient in octanol/water, hexadecane/water, and cyclohexane/water.

MDFP was also used by Gebhardt et al. (2020). In this approach, ML was combined with the atomistic MD simulations encoded with MDFPs enabling the large-scale free-energy calculations. The so-called ML/MDFP method overcomes limitations related to free-energy estimation with MD – high computational expense and imperfections of force-fields. ML models are able to detect systematic force field errors caused by specific chemical groups and, afterwards, decrease their influence on final prediction. Moreover, ML models provide efficient and fast calculations when working with fingerprints databases; as an example, Gebhart et al. utilized the distributions of potential energy of the solute, radius of gyration, and SASA, which were generated from MD data. The outcomes proved that ML/MDFP approach predicted free-energy not worse or even slightly better than rigorous free-energy simulations and two models, namely quantum chemistry-based COSMO-RS. When two models for free energy predictions (COSMO-RS and UNIFAC) were compared with the support vector regression (SVR), it appeared that the latter one demonstrated the best results. The other application of fingerprints extracted from MD could be distinguishing active compounds, as Jamal et al. (2019) proved on the example of caspase-8 ligands. MD descriptors determined in this work were analogous to those obtained by Gebhardt et al. Moreover, fingerprints of different types were also calculated for reference. Multiple combinations of 2D, 3D, and MD descriptors were used to train two ML models: artificial neural networks and Random Forest. MD descriptors used individually showed better performance than being combined with other 2D/3D descriptors, which proved applicability of MD descriptors for lead prioritization and optimization of caspase-8 ligands.

Ash and Fourches (2017) made benefits of combination of MD and chemical descriptors to generate innovative QSAR models based on MD data, resulting in the construction of the so-called hyperpredictive MDQSAR models. The researchers in their work hypothesized that exploring dynamic noncovalent protein-ligand interactions would help to distinguish active compounds from non-active. A set of ERK2 inhibitors served as a case study, after previous unsuccessful attempts to rank them using conventional QSAR and sophisticated molecular docking techniques. Each ligand was docked in the ERK2 binding site using Glide, then 20 ns simulations of obtained ligand-protein complexes were performed in Desmond. MDs were followed by the extraction of descriptors on the basis of MD data with KNIME, such as traditional 1D-MACCS fingerprints, as well as 2D RDKit, 3D-D Moments and 3D-WHIM descriptors. The results indicate that MD descriptors successfully tackled the primary challenge and clearly pointed out the most active ligands. The hierarchical clustering highlighted similarities between MD descriptors and activities; furthermore, MD descriptors turned out to be useful in the identification of activity cliffs in all descriptor spaces. The research underlines the importance of further investigation of the MD descriptors usage, which could lead to implementation of new highly effective MDQSAR models in the future computer-aided drug design workflows.

MD data were also used by Vitek et al. (2013) to develop Support Vector Regression (SVR) model for water molecule energy estimation and by Jamroz et al. (2012) to examine fluctuations of protein residues during simulation.

Exploring protein conformations is extremely useful in understanding protein structure and function. However, to capture conformational changes we would need to perform long-time simulations and overcome multiple high energy barriers between local energy minima, which is related to the consumption of significant amounts of computational resources. Traditionally, enhanced sampling methods are exploited to solve these problems; however, their efficiency requires improvement (Yang et al., 2019). Fortunately, owing to technology advances, numerous novel efficient techniques have been developed. For example, a number of DL-based, approaches have already been proposed, such as variational autoencoders (VAEs), which significantly increases sampling “power”, if combined with MD potential. Tian et al. (2021) demonstrated successful protein sampling with VAEs on the example of adenosine kinase (ADK) conformational change from its closed state to the open one. Decoded conformations were similar to the training ones. Additionally, the latent space provided by VAEs could serve as a starting point for new simulations and studying of unexplored conformational spaces. VAEs application allows to perform short simulations of 20 ns and reach sampling efficiency comparable to a single long MD simulation. Another example of analysis of MD trajectories of proteins applies the Bayesian interference method to perform structural fitting for removing time-dependent translational and rotational movements (Miyashita and Yonezawa, 2017). On the other hand, Perez et al. (2015) combined MD with Bayesian interference to speed up simulation. The combination of Bayesian interference with MD simulations was also used by Shevchuk and Hub (2017) to refine structures and ensembles against small-angle X-ray scattering (SAXS) data.

Proteins change their conformations upon the influence of many factors, such as temperature, pH, and more importantly as a consequence of molecular recognition due to ligand binding (Doms et al., 1985; Takeda et al., 1989; Andersen et al., 1990). What is more, the ligand-protein complex is formed by the induced fit of both molecules, and the resulting protein conformations depend on the structure of the ligand (Bosshard, 2001). Conformational dynamics of proteins have a profound effect on cell functioning, such as in the case of G-protein coupled receptors (GPCRs), which transduce external signals into cells by activation of specific cellular pathways. The binding of different ligands stabilizes certain conformational state, which results in the elicitation of distinct signalling—a phenomenon called functional signalling, or biased agonism (Hilger et al., 2018; Wootten et al., 2018). An essential role of GPCRs in signal transmission highlights the importance of understanding how ligand binding alters protein conformations, in order to design new GPCR ligands, which would target desired pathways and avoid others, potentially causing side effects. MD is perfectly suited for perceiving ligand-protein conformational change; however, the difficulty lies in the necessity to analyze long-scale MD simulations, which are required to capture tiny structural changes, responsible for functional signalling. Plante et al. (2019) successfully applied deep neural networks (DNNs), to analyze MD data. MD output was transformed into the pixel representation, which is interpretable by the state-of-art DL object-recognition technology. When the method was applied to the pharmacological classification of 5-HT_2A_ and D_2_ receptors ligands, among which were full, partial, and inverse agonists, DNN achieved near-perfect accuracy, classifying correctly >99% frames. Moreover, the sensitivity analysis identified the molecular determinants, which were considered by the model as the most important for the correct prediction. Even if the study has limited scope, including only eight ligands and two receptors, it gives hope for the highly accurate and efficient estimation of ligand-protein functional selectivity with the help of DNN.

Allostery is called the second secret of life (Fenton, 2008), as it is crucial for the adaptation of living organisms to changing environmental conditions by altering multiple cell functions, like enzyme catalysis, cell signalling, gene transcription, and others (Goodey and Benkovic, 2008; Nussinov et al., 2014). Designing allosteric drugs is a challenging task for multiple reasons. First of all, classical docking alone is unable to predict how orthosteric binding sites would adjust to allosteric modulation, and, importantly, which functional effect ligands would exert on protein’s function (Nussinov and Tsai, 2013; Lu et al., 2019; Sheik et al., 2020). Luckily, MD simulations give insight into the nature of allosteric perturbations; moreover, the application of ML algorithms to MD data expands possibilities to extract valuable information from long-scale simulations. Recently conducted research proved that such a combined MD-ML approach is able to efficiently determine ligand’s functional activity and models explaining ligand efficacy can be constructed. Marchetti et al. (2021) brought together the benefits of ensemble docking, MD and ML, in order to predict whether a set of ligands would inhibit or activate molecular chaperone Hsp90. MD of Hsp90 with several ligands was followed by cluster analysis of the obtained metatrajectory, subsequently, representative protein conformations were chosen for ensemble docking. The features obtained from docking, notably docking score, RMS, and RMSD, were used for training a supervised model, which served as a classification tool. Among three popular algorithms—logistic regression, SVM, and Random Forest - SVM reached the highest accuracy (0.9), as well as showed the best performance. On the other hand, attempts to classify ligands on the basis of separate features or chemometrics properties (here, molecular fingerprints) were far less efficient. In contrast, Ferraro et al. (2021) aimed to predict allosteric ligand functionality quantitatively. A computational experiment was performed on the allosteric modulators of the molecular chaperone TRAP1, which had similar affinities, but inhibited ATPase function with different efficacy. Two ML algorithms–Naïve Bayes and SVM–were applied to extract the local dynamic patterns responsible for the allosteric perturbation. The models were trained and validated on MD simulations of the perturbed and unperturbed systems. Whereas the discriminative SVM models qualitatively assessed the disparities between the perturbed and unperturbed ensembles, the implementation of the generative Naïve Bayes model produced a linear regression model with a 0.71 correlation between predicted states in the inhibitor-bound trajectories (TPR percentage) and the TRAP1 inhibition percentage. Additionally, Naïve Bayes could estimate the weight of ligand effects on each feature, which would support the identification of the features crucial for the allosteric propagation. Therefore, ML expands the possibilities of computer-aided drug design of allosteric modulators and could bring drug design to a new level with limited experimental testing.

The number of proteins with unknown functions is increasing due to the advances in bioinformatics, especially in the field of structural genomics. Identification of binding pockets could potentially be the key to understanding which functions specific proteins carry out. The FEATURE (Wei and Altman, 1998) is an ML-based algorithm for the identification of Ca^2+^-binding sites, utilizing the Bayesian scoring scheme. The FEATURE prediction does not depend on the sequence or structure, as the models examine local 3D physicochemical environment and that is why they are able to recognize diverse binding sites. However, the applications of the algorithm were limited to static structures, until Glazer et al. (2008) applied MD to improve the FEATURE detection ability by increasing structural diversity. The hypothesis was tested on parvalbumin β – an EF-hand Ca^2+^-binding protein, which has two Ca^2+^-binding sites–and MD-assisted calcium-binding pockets recognition. Moreover, relatively small time steps were characterized by significant change in the FEATURE scores, meaning that the FEATURE is very sensitive to small conformational changes, which might have an impact on calcium binding. These promising results could help to implement MD methodology in the exploration of protein functions.

Researchers’ efforts and technological advancement resulted in the development of a framework designed to support performing of MD simulations by means of ML algorithms – TorchMD (Doerr et al., 2021). Since the toolset is written in PyTorch (Paszke et al., 2019), it can be easily integrated with other models from this ML library. Among essential features of the framework is TorchMD-Net, which takes advantage of training neural network potential in order to improve force-field development. Furthermore, TorchMD enables running simulations with end-to-end differentiability of parameters, beneficial for the performance of steered and highly constrained MD simulations, sensitivity analysis, and others. Additionally, TorchMD with implemented neural network potential is used for coarse-grained MD simulations, which are helpful in studying protein folding and exploring conformational space. Code, step-by-step tutorials, and data are available at GitHub (https://www.github.com/torchmd).

## Conclusion

Both intense growth in the amount of data, as well as increasing capabilities of various algorithms to detect patterns and relationships in various sets of information, dramatically increased the popularity of automatic approaches for MD outcome analysis. The output of such experiments consists of billions of timesteps, and recorded positions and velocities of thousands of atoms. Therefore, extracting important information from such a data package can be very challenging, and so the application of various post-processing approaches is needed. The post-processing protocols can help in the finding of non-obvious ligand-protein interaction patterns, detection of rare conformational states, or examining dependence of conformational changes of the examined system in time. Moreover, thanks to the post-processing approaches, the prediction of the system behavior in longer time scales than modeled can be made.

However, given all the advantages of ML approaches, we should still be aware of their limitations and pay attention to data used for models training, as it will substantially define the quality of the outcome. Importantly, ML models could have limited transferability and must be applied to other types of data carefully. Nevertheless, application of ML to MD data is undoubtedly the future, which makes the potential of MD applications almost unlimited.
